# Persistent Voice Changes Following Total Thyroidectomy Despite Intraoperative Nerve Monitoring: A Retrospective Single-Center Study

**DOI:** 10.7759/cureus.104693

**Published:** 2026-03-05

**Authors:** Nufra Senofer, Adil Mohammed Suleman, Vinod Kumar Singhal, Adel Mohamed Yasin Alsisi

**Affiliations:** 1 ENT, Prime Hospital, Dubai, ARE; 2 General Surgery, Prime Hospital, Dubai, ARE; 3 Intensive Care, Prime Hospital, Dubai, ARE

**Keywords:** hoarseness, intraoperative nerve monitoring, postoperative complications, recurrent laryngeal nerve, thyroid surgery, total thyroidectomy, voice changes

## Abstract

Objective: This study aimed to determine the incidence of postoperative voice changes following total thyroidectomy and to characterize these changes in patients with documented intact intraoperative nerve monitoring (IONM), while comparing outcomes between surgeries performed with and without IONM.

Methodology: This single-center retrospective observational study included 480 patients who underwent total thyroidectomy between 2015 and 2025. Among them, 350 patients had surgery performed with IONM, while 130 underwent the procedure without it. Postoperative voice changes were documented, and all patients with voice complaints underwent laryngoscopic evaluation to assess vocal cord mobility and exclude recurrent laryngeal nerve (RLN) injury. Descriptive statistics and comparative analyses were used to evaluate correlations within the data.

Results: Postoperative voice alterations were reported in 4 (0.83%) patients, all of whom were in the IONM group. In each case, intraoperative and postoperative assessments confirmed no injury to the RLN. These voice changes resolved spontaneously within 3-4 months without specific intervention. No patients in the non-IONM group reported any voice complaints. The difference in the prevalence of postoperative voice change between the IONM and non-IONM groups was not statistically significant (p > 0.05).

Conclusion: This study demonstrated a low incidence of symptomatic postoperative voice changes following total thyroidectomy, with transient dysphonia resolving spontaneously in all affected patients. The occurrence of voice changes in the IONM group was not statistically significant and likely reflects differences in surgical complexity rather than the effect of monitoring itself. Larger prospective multicenter studies incorporating standardized objective voice assessments are warranted to clarify postoperative voice outcomes further.

## Introduction

Voice changes following total thyroidectomy are well-recognized postoperative complications and represent a significant cause of morbidity among patients undergoing thyroid surgery. These changes may manifest as hoarseness, vocal fatigue, breathy voice, reduced vocal range, or even dysphonia [1]. Although these symptoms are often transient, they may persist for several months and, in some cases, become permanent, significantly affecting a patient’s personal and professional life [2].

The recurrent laryngeal nerve (RLN) and the external branch of the superior laryngeal nerve (EBSLN) are particularly vulnerable during thyroid surgery because of their close anatomical proximity to the thyroid gland [3]. Injury to these nerves, whether partial or complete, can result in substantial vocal dysfunction. To reduce this risk, intraoperative nerve monitoring (IONM) has been increasingly employed as an adjunct to direct visualization. IONM allows real-time identification and functional verification of the RLN and EBSLN during surgery, thereby minimizing the likelihood of accidental injury [4,5].

Despite the routine use of IONM, some patients still report postoperative voice changes even when no nerve injury is detected [6]. These cases are often classified as idiopathic or non-neurogenic in origin and may result from transient neuropraxia, trauma from endotracheal intubation, laryngeal edema, traction injury, or psychological factors [7]. The occurrence of such symptoms despite preserved intraoperative nerve function highlights the limitations of IONM and the complex nature of voice physiology [8].

Furthermore, discrepancies between objective nerve integrity and subjective voice complaints pose diagnostic challenges and may lead to patient dissatisfaction. This underscores the need for further investigation into the true incidence, characteristics, and potential etiologies of postoperative voice changes in thyroidectomy patients with intact IONM results [9].

The primary objective of this retrospective single-center study was to determine the incidence of postoperative voice changes in patients undergoing total thyroidectomy between January 2015 and January 2025. The secondary objectives were to characterize the nature and duration of postoperative voice changes in patients with documented intact IONM findings, including preserved RLN and EBSLN function, and to compare the incidence of postoperative voice changes between patients who underwent surgery with IONM and those without IONM. Postoperative voice change was defined as hoarseness, pitch alteration, or vocal fatigue persisting for more than two weeks, based on retrospective clinical documentation and follow-up records of at least three months.

## Materials and methods

Study design and setting

This 10-year, single-center retrospective observational study was conducted in the Department of Endocrine Surgery at Prime Hospital, Dubai, United Arab Emirates, from January 2015 to January 2025. All patients who underwent total thyroidectomy for benign or malignant thyroid disease were included. The study aimed to evaluate the incidence of postoperative voice changes, particularly among patients who underwent IONM and in whom no nerve damage was documented. All surgeries were performed at a tertiary-care private hospital with a dedicated endocrine surgery unit. A standardized surgical approach (conventional open total thyroidectomy via collar incision) was used. Experienced endocrine surgeons performed procedures in accordance with uniform institutional protocols.

Routine identification and careful dissection of the RLN and EBSLN were performed under direct visualization in all cases. Hemostasis was achieved using conventional ligation techniques and energy-based devices, in accordance with institutional safety guidelines.

Selection criteria

Patients were eligible if they were aged 18 years or older, underwent total thyroidectomy between January 2015 and January 2025, and had complete intraoperative and postoperative data, including at least three months of follow-up. The three-month follow-up cutoff was selected to differentiate transient postoperative dysphonia from early functional changes and to align with commonly accepted definitions of temporary versus persistent nerve dysfunction in thyroid surgery literature. Patients were routinely evaluated postoperatively at two weeks, three months, and six months when indicated. In the IONM group, inclusion required intraoperative verification of the integrity of both the RLN and the EBSLN.

Exclusion criteria were preexisting voice disorders or vocal cord paralysis, incomplete medical records, conversion from subtotal to total thyroidectomy during surgery, and loss to follow-up before the third postoperative month.

Sample size

A total of 480 cases were included in the final analysis. Among them, 350 patients underwent surgery with IONM, while 130 did not. The sample size was determined based on the availability of complete operative and follow-up data within the 10-year study period.

Data sources and variables

Data were extracted from hospital medical records, surgical logs, and postoperative ENT evaluations. The primary outcome was postoperative voice change, defined as hoarseness, pitch alteration, or vocal fatigue persisting for more than two weeks. Severity was categorized descriptively as mild (voice fatigue without functional limitation), moderate (persistent hoarseness affecting daily communication), or severe (voice impairment requiring formal ENT evaluation or intervention), based on clinical documentation.

Voice assessment was performed clinically and, when indicated, by indirect laryngoscopy. Laryngoscopy was performed in all patients who reported persistent voice symptoms beyond two weeks postoperatively. Assessments were conducted by an ENT specialist. No standardized acoustic analysis tools (e.g., jitter, shimmer, or validated questionnaires) were routinely used, reflecting the retrospective nature of the study.

Intraoperative variables included IONM use, verification of nerve integrity, surgery duration, and thyroidectomy indication (benign or malignant). IONM was performed using a standardized intermittent stimulation protocol with endotracheal tube-based surface electrodes.

Baseline electromyographic signals were obtained after endotracheal intubation. Intermittent stimulation of the vagus nerve and RLN was performed before and after thyroid gland removal using manufacturer-recommended current settings. Nerve integrity was confirmed by preservation of a stable and reproducible electromyographic response following stimulation, in addition to direct visual identification.

All surgeries followed a consistent nerve identification and preservation technique. Energy devices were used according to the surgeon’s preference, but within institutional safety standards, and direct visualization of the RLN and EBSLN was mandatory in all cases.

Additional data included patient demographics, comorbidities, anesthesia details, and follow-up duration. Anesthesia records were reviewed; however, specific intubation-related variables, such as tube size, cuff pressure monitoring, intubation duration, and intubation difficulty, were not consistently documented and therefore could not be analyzed.

All patients underwent general anesthesia with endotracheal intubation according to standardized institutional anesthesia protocols.

Statistical analysis

Data were compiled using Microsoft Excel (Microsoft Corp., Redmond, WA, USA) and analyzed with IBM SPSS Statistics for Windows, Version 25.0 (Released 2017; IBM Corp., Armonk, NY, USA). Descriptive statistics were used to summarize demographic and clinical data. Categorical variables, such as the incidence of voice change, were expressed as frequencies and percentages, while continuous variables, such as age, were presented as mean ± standard deviation. The chi-square test or Fisher’s exact test was applied to compare the rate of postoperative voice changes between the IONM and non-IONM groups. A p-value < 0.05 was considered statistically significant.

## Results

Table 1 presents the distribution of patients according to the use of IONM during total thyroidectomy. Among 480 patients, IONM was employed in 350 (72.9%) cases, while 130 (27.1%) underwent surgery without it. 

**Table 1:** Distribution of patients based on IONM usage IONM: intraoperative nerve monitoring.

Category	Number of patients	Percentage (%)
IONM used	350	72.9%
IONM not used	130	27.1%
Total	480	100%

Table 2 presents the incidence of postoperative voice changes in the study cohort. Of 480 patients, 4 (0.83%) experienced transient postoperative voice changes, whereas 476 (99.17%) reported no alterations. All transient cases resolved spontaneously within 3-4 months, underscoring the overall safety of total thyroidectomy with respect to laryngeal function.

**Table 2:** Incidence of postoperative voice changes

Voice changes postoperatively	Number of cases	Percentage (%)
Yes	4	0.83%
No	476	99.17%
Total	480	100%

Among 350 patients who received IONM, 4 (1.14%) developed transient voice changes, while none were reported among the 130 patients without IONM. Although this difference appeared notable, it was not statistically significant (p > 0.05), indicating that IONM was not independently associated with a reduced incidence of transient hoarseness. Table 3 summarizes the clinical characteristics of the four patients who experienced postoperative voice alterations. All were female except one male, and all underwent surgery with IONM. No intraoperative evidence of recurrent RLN or EBSLN injury was documented. Voice changes were temporary and resolved within 3-4 months without additional intervention. 

**Table 3:** Details of voice change cases (n = 4) IONM: Intraoperative nerve monitoring, RLN: recurrent laryngeal nerve, EBSLN: external branch of the superior laryngeal nerve

Case no.	Age	Gender	IONM used	RLN injury	EBSLN injury	Recovery duration	Additional findings
1	42	Female	Yes	None	None	3 months	Mild voice fatigue initially
2	58	Male	Yes	None	None	4 months	Transient breathy voice
3	36	Female	Yes	None	None	3.5 months	No anatomical abnormalities
4	45	Female	Yes	None	None	3 months	Voice therapy not required

## Discussion

Postoperative voice change following total thyroidectomy is a well-recognized complication that contributes significantly to patient morbidity. Thyroid disorders are common worldwide, with thyroid nodules affecting up to 50%-60% of the adult population on ultrasonography and thyroid cancer representing one of the most frequently diagnosed endocrine malignancies globally. Consequently, total thyroidectomy is a commonly performed surgical procedure, making postoperative functional outcomes, including voice changes, clinically important [1]. Postoperative dysphonia is most often attributed to injury or neuropraxia of the RLN or the EBSLN [10]. Despite advances in surgical techniques and the increasing use of IONM, transient dysphonia continues to occur, even in cases without evident nerve trauma. This underscores the multifactorial nature of postoperative voice disorders, which may arise from factors beyond nerve injury, including endotracheal intubation trauma, laryngeal edema, traction, or temporary alterations in voice mechanics [6,11].

In the present study involving 480 patients who underwent total thyroidectomy over 10 years, 350 surgeries (72.9%) were performed with IONM, and 130 (27.1%) without it. Transient voice changes were noted in four patients, all of whom were within the IONM group. None of these patients showed intraoperative evidence of nerve injury, and all cases resolved spontaneously within 3-4 months. No voice changes were reported among patients in the non-IONM group, though this difference was not statistically significant.

These findings align with previous reports suggesting that while IONM is effective in identifying and preserving nerve function, it does not eliminate postoperative dysphonia [12,13]. Barczyński et al. [14] demonstrated that IONM reduced the rate of RLN palsy compared with visual identification alone, yet temporary nerve dysfunction persisted in 1.9% of patients despite intact anatomy. Similarly, Staubitz et al. [15] observed that postoperative voice changes may occur despite confirmed intraoperative nerve integrity, likely due to neuropraxia or soft-tissue manipulation. Such cases are typically transient, consistent with our findings that reflect functional rather than structural impairment.

The temporary nature of dysphonia in our patients points toward probable causes such as traction injury, thermal spread, ischemia, or intubation-related laryngeal irritation. Supporting this, Huang et al. [16] noted that even with preserved electromyographic activity of the RLN, postoperative voice disturbances may result from non-neurological causes. The absence of permanent nerve palsy in our series reinforces the safety of current surgical techniques and highlights the role of IONM in minimizing major complications.

Strengths and limitations

A key strength of this study is its large sample size and extended 10-year follow-up, which allow meaningful evaluation of postoperative voice outcomes following total thyroidectomy. The use of standardized surgical techniques within a dedicated endocrine surgery unit and consistent institutional follow-up protocols enhances the internal validity of the findings. Additionally, a high proportion of patients underwent IONM, reflecting contemporary surgical practice.

However, several limitations must be acknowledged. First, as a retrospective study, the analysis depended on available clinical documentation, which may have led to underreporting of mild or transient voice symptoms. Although patients with persistent symptoms underwent laryngoscopic evaluation by an ENT specialist, standardized acoustic voice analysis tools, such as jitter and shimmer, or validated perceptual scales were not routinely used. This limits the objective quantification of subtle voice changes and may affect comparability with studies that employ formal acoustic assessment. Second, while a descriptive clinical grading of voice severity was applied based on documentation, no validated severity scoring system or structured questionnaire was used. Future prospective studies incorporating standardized voice outcome measures would enhance reproducibility.

Third, anesthesia-related variables that may influence postoperative voice outcomes, such as endotracheal tube size, cuff pressure monitoring, duration of intubation, and intubation difficulty, were not consistently recorded in operative records and therefore could not be analyzed. These factors may contribute to non-neurogenic dysphonia and represent potential confounders. Fourth, although a standardized open surgical approach and institutional nerve identification protocol were followed, minor variations in surgical technique and energy device usage between surgeons cannot be entirely excluded.

Finally, the three-month follow-up threshold was selected to differentiate transient from early postoperative voice changes; however, some voice parameters may persist beyond this period. Longer-term follow-up with a structured assessment would provide additional insight into persistent functional alterations.

Despite these limitations, the study provides valuable real-world data on postoperative voice outcomes following thyroidectomy, particularly in patients with documented intact nerve integrity.

## Conclusions

This single-center retrospective study demonstrates a low documented incidence of symptomatic postoperative voice changes following total thyroidectomy. Transient dysphonia was observed in a small proportion of patients, including cases in which IONM confirmed preserved nerve integrity. All reported cases resolved spontaneously within a few months, suggesting a predominantly temporary functional disturbance. However, given the retrospective design, the absence of a universal, standardized acoustic voice assessment, potential unmeasured confounders, and variability in follow-up duration, the findings should be interpreted with caution. The observation that voice changes occurred only in the IONM group was not statistically significant. It may reflect differences in case selection, surgical complexity, or other unmeasured factors rather than a direct effect of monitoring itself. While IONM remains an important adjunct for nerve identification and preservation, this study does not allow definitive conclusions regarding its impact on postoperative voice outcomes. Prospective multicenter studies incorporating standardized objective voice assessments and controlled perioperative variables are warranted to better clarify the true incidence, mechanisms, and risk factors for postoperative dysphonia.
